# Group B *Streptococcus* colonization induces *Prevotella* and *Megasphaera* abundance-featured vaginal microbiome compositional change in non-pregnant women

**DOI:** 10.7717/peerj.7474

**Published:** 2019-08-16

**Authors:** Xiaofeng Mu, Changying Zhao, Junjie Yang, Xiaofang Wei, Jiaming Zhang, Cheng Liang, Zhongtao Gai, Chunling Zhang, Dequan Zhu, Ye Wang, Lei Zhang

**Affiliations:** 1 Tianjin University, Academy of Medical Engineering and Translational Medicine, Tianjin, China; 2 Clinical Laboratory and Core Research Laboratory; Qingdao Human Microbiome Center & Qingdao Institute of Oncology, The Affiliated Central Hospital of Qingdao University, Qingdao, China; 3 Beijing Advanced Innovation Center for Big Data-Based Precision Medicine, Beihang University, Beijing, China; 4 Shandong Children’s Microbiome Center, Qilu Children’s Hospital of Shandong University, Jinan, China; 5 Research Institute of Pediatrics, Qilu Children’s Hospital of Shandong University, Jinan, China; 6 College of Life Science, Qilu Normal University, Jinan, China; 7 School of Basic Medical Sciences, Shandong University, Jinan, China; 8 School of Information Science and Engineering, Shandong Normal University, Jinan, China; 9 School of Precision Instruments and Optoelectronics Engineering, Tianjin University, Tianjin, China; 10 Microbiological Laboratory; Department of Infection Management; Department of Neurosurgery, Lin Yi People’s Hospital, Linyi, China

**Keywords:** Vaginal microbiome, Group B Streptococcus, Microbial colonization, Disease transmission

## Abstract

**Background:**

Previous studies have indicated that variations in the vaginal microbiome result in symptomatic conditions. Group B *Streptococcus* (GBS) is a significant neonatal pathogen and maternal vaginal colonization has been recognized as an important risk factor for neonatal disease. Therefore, it is important to discover the relationship between the composition of the vaginal microbiome and GBS colonization. This study explores the potential relationship between the composition of the vaginal microbiome and GBS colonization in non-pregnant Chinese women.

**Methods:**

A total of 22 GBS-positive, non-pregnant women and 44 matched GBS-negative women were recruited for the current study. The composition of the vaginal microbiome was profiled by sequencing the 16S rRNA genes. The microbiome diversity and variation were then evaluated.

**Results:**

The vaginal microbiome of the 66 subjects enrolled in the current study were compared and the results showed that GBS-positive women exhibited significant vaginal microbial differences compared with the GBS-negative women based on the analysis of similarities (*r* = 0.306, *p* < 0.01). The relative abundance of the bacterial genus *Lactobacillus* (*p* < 0.01) was significantly lower in the GBS-positive group, while the abundances of the bacterial genera *Prevotella* (*p* < 0.01), *Megasphaera* (*p* < 0.01), and *Streptococcus* (*p* < 0.01) were significantly higher in the GBS-positive group.

**Discussion:**

The current study addressed significant variations across the communities of the vaginal microbiome in GBS-positive and GBS-negative women in a Chinese cohort, which paves the way for a larger cohort-based clinical validation study and the development of therapeutic probiotics in the future.

## Introduction

Group B *Streptococcus* (GBS) is a Gram-positive bacteria that asymptomatically and transiently colonizes the gastrointestinal and vaginal tracts of healthy women. It is the leading cause of invasive bacterial disease in infants and can lead to a fatal infection in newborns (Korir, Manning & Davies, 2017). There are more than three million neonatal deaths worldwide each year, 20% of which are caused by infections, which is a global concern (Rick et al., 2017). Since the 1970s, GBS has been considered to be the main cause of perinatal infection. GBS colonization in infants less than 7-days old can cause septicemia and pneumonia, while in infants 7 days to 3 months of age, bacteremia and meningitis are often the major clinical complications of GBS colonization (Zaleznik et al., 2000). GBS is thought to contribute to chorioamnionitis, endometritis, urinary tract infections, sepsis, and premature birth in peripartum women (Krohn, Hillier & Baker, 1999; Yancey et al., 1994).

Since GBS is the leading cause of neonatal morbidity and mortality, several rapid tests based on molecular techniques and therapies for GBS have been developed and applied clinically (Davies et al., 2004). Pregnant women need to be screened between 34 weeks and 37 weeks of pregnancy and GBS-positive pregnant women should be considered for antibiotic treatment to protect newborns from GBS infection. Many antibiotics are widely believed to have an effect on newborns (Puopolo, Madoff & Eichenwald, 2005), therefore, probiotic therapy is currently considered to be an important adjuvant during antibiotic treatment. The proper long-term use of probiotics has not been found to negatively affect non-pregnant women and fetuses (Lundelin et al., 2017). The development of probiotic intervention strategies in the era of precision medicine relies on extensive research of the vaginal microbiome.

Previous studies have mainly examined the characteristics of GBS colonization in pregnant women. Vaginal bacterial cultures were taken from 4,025 women at 22 to 36 weeks of gestation and a significantly lower likelihood of coagulase-negative *Staphylococcus*, *Prevotella,* and *Lactobacillus* was found in the GBS-positive group (Kubota, Nojima & Itoh, 2002). It was observed that colonization by GBS could change the vaginal microbiome. GBS detection in healthy pregnant women correlated with a decrease in the *Lactobacillus* species (Altoparlak, Kadanali & Kadanali, 2004). In another study, there were no significant differences in the *Lactobacillus* or *Bifidobacterium* numbers based on GBS colonization (Brzychczy-Wloch et al., 2014). Recently, a study of 428 women in the USA showed that *alpha* diversity was not related to GBS status (Rosen et al., 2017). The discrepancy in the quantity of GBS-related taxa mentioned in these studies is probably due to different sampling locations, patient demographics, and sequencing strategies. Additionally, the relationship between GBS colonization and the vaginal microbiota can be affected by complex factors including education, ethnicity, geography, sexual behavior, and socioeconomic class (Albert et al., 2015). The previous studies mainly used culture-based methods to examine the relationship between GBS colonization and other components of the vaginal bacteria (Aveyard et al., 2002). However, this method often shows bacterial phenotypic expression instability and does not comprehensively reveal the relationship between GBS colonization and the microbiome in the female reproductive tract. The majority of the previous studies were also aimed at pregnant women (Kubota, Nojima & Itoh, 2002; Altoparlak, Kadanali & Kadanali, 2004). There were relatively few studies on non-pregnant women and especially few studies on the Asian cohort, which made GBS invasive bacterial disease in infants difficult to determine without adequate references. Therefore, we performed a pilot study to evaluate the potential relationship of the composition of the vaginal microbiome with GBS colonization in non-pregnant Chinese women.

## Materials & Methods

### Participants and study design

The current study was carried out in accordance with the recommendations of the Human Specimen Study Guidelines of the Institutional Review Board of the Affiliated Central Hospital of Qingdao University (IRB# QCH17-0328-01). Written informed consents were obtained from all patients before they were randomly assigned to groups. This research was conducted in compliance with national legislation and the Code of Ethical Principles for Medical Research Involving Human Subjects from the World Medical Association (Declaration of Helsinki). Women aged 25–42 years seeking primary gynecologic care were enrolled from March 2017 to December 2017 at the Qingdao Medical Center (Table 1). Over 600 samples were collected from individuals with GBS colonization and healthy individuals and vaginal samples for the current microbiome study were chosen from this specimen bank. The inclusion criteria for the current study were: (a) Women between the ages of 25–42 years, (b) non-pregnant, (c) sexually active, (d) HIV negative, (e) without clinically evident inflammatory conditions, and (f) women who didn’t use any oral or intravaginal antibiotic or antifungal medications for three months prior to the sample collection (Figure S1).

**Table 1:** Characteristics of study participants.

	GBS-P (*n* = 22)	**GBS-N (*n* = 44)**	*P*-value
Age (year)	29.86 ± 3.76	30.15 ± 4.02	0.7662
Parity			0.8153
primiparity	18 (81.82%)	37 (84.10%)	
multiparity	4 (18.18%)	7 (15.91%)	
Use of hormonal contraceptives	2(9.10%)	3(6.82%)	0.8694
History of bacterial vaginitis	5(22.73%)	8(18.18%)	0.6616
Vaginal cleanliness			0.5873
I–II grade	13(59.09%)	29(65.9%)	
III–IV grade	9(40.91%)	15(34.1%)	

A GBS nucleic acid detection kit (BioChain, Beijing, China) was used to conduct preliminary testing for GBS colonization. Out of a total of 66 women screened, 22 women were GBS-positive and the other 44 were GBS-negative. Vaginal swab samples were collected, processed, and transferred to −80 °C storage within 30 min. In order to prevent contamination, strict standards and procedures were adopted. Sexual intercourse, tub bath, vaginal examination, vaginal lavage, and localized medication were prohibited 24 h before the collection of the vaginal specimens in order to avoid affecting the results of the examination. Sterilized scrapers, straws, or cotton swabs used for collecting samples were clean and dry without any chemicals or lubricants. The vaginal speculum could be moistened with a little saline, if necessary, before insertion. The swabs were placed directly into the sterile container.

### Microbial DNA extraction, 16S rRNA gene amplicon sequencing

DNA was isolated from the vaginal swabs using the QIAamp Fast DNA Stool Mini Kit (Qiagen, Valencia, CA, USA) (Sundquist et al., 2007). The DNA concentration was measured by NanoDrop ND-2000 (Thermo Fisher Scientific, Waltham, MA, USA). The V1–V2 hyper-variable region of the bacterial 16S rRNA gene was identified to analyze the microbial community within the samples. Two universal bacterial 16S rRNA gene amplicon PCR primers (PAGE purified) were used: forward primer-27F (5′AGAGTTTGATCMTGGCTCAG3′) and reverse primer-355R (5′GCTGCCTCCCGTAGGAGT3′). Amplicons were then purified using the QIAquick PCR Purification Kit (Qiagen) PCR purification procedure, quantified using a NanoDrop ND-1000 Spectrophotometer (Nucliber, Madrid, Spain), and then pooled in equal concentrations. All amplicons were quantified and pooled to equalize concentrations for sequencing using HiSeq 2500 (Illumina, San Diego, CA, USA).

### Sequence processing and statistical analysis

The 16S rRNA gene sequence paired-end data set was merged and filtered using the FLASH method described by Magoč and Salzberg (Magoc & Salzberg, 2011). All sequence analysis was conducted by the Quantitative Insights into Microbial Ecology (QIIME, version 1.9.1) software suite (Caporaso et al., 2010), as per the QIIME tutorial (http://qiime.org/). Chimeric sequences were removed using usearch61 (Edgar, 2010) with *denovo* models. Sequences were clustered against the 2013 Greengenes (13_8 release) ribosomal database’s 97% reference data set (http://greengenes.secondgenome.com/?prefix=downloads/greengenes_database/).

Sequences that did not match any entries in this reference were subsequently clustered into de novo OTUs at 97% similarity with UCLUST. Taxonomy was assigned to all OTUs using the RDP classifier (Cole et al., 2009) within QIIME and the Greengenes reference data set.

OTU tables were subsequently subjected to abundance-based filtering, removing low-abundance OTUs that represented less than 0.005% of total reads in the data set. The OTU table was then rarefied to a sequencing depth of 25000 per sample, for subsequent analyses of *alpha* and *beta* diversity. *Alpha* diversity (observed OTUs and Shannon) and *beta* diversity (PCoA analysis) were analyzed using the QIIME standard pipeline.

### Statistical analysis

The data were presented as the mean ± SD. The chi-squared-test was used to assess gender differences, and the Kolmogorov–Smirnov test was used to evaluate *alpha* diversity. An analysis of similarities (ANOSIM) on *beta* diversity matrices was conducted in QIIME to test for significant differences between the microbial communities. The significance of the ANOSIM test was assessed with 999 permutations. Linear discriminant analysis effect size (LEfSe) was introduced to identify bacterial biomarkers for two groups and was performed on the web-based Galaxy interface (http://huttenhower.sph.harvard.edu/galaxy) (Segata et al., 2011). Standard parameters were used, except for the alpha value for the factorial Kruskal-Wallis test among classes (alpha < 0.01) and the threshold used to consider a discriminative feature for the logarithmic LDA score was set at >3. Statistical dependence between continuous variables was determined using a Spearman’s rank correlation.

## Results

Vaginal swab samples were collected from 66 female patients who met the inclusion criteria, which included 22 GBS-positive women and 44 GBS-negative women. The clinical characteristics of the subjects are summarized in Table 1. The age of the subjects ranged from 25 to 42 years, with an average age of 31.40  ± 4.05 years (GBS-negative group, 32.15 ± 4.02; GBS-positive group, 29.86 ± 3.76).

### Different composition of vaginal microbiome in GBS-positive female patients

**Figure 1:**
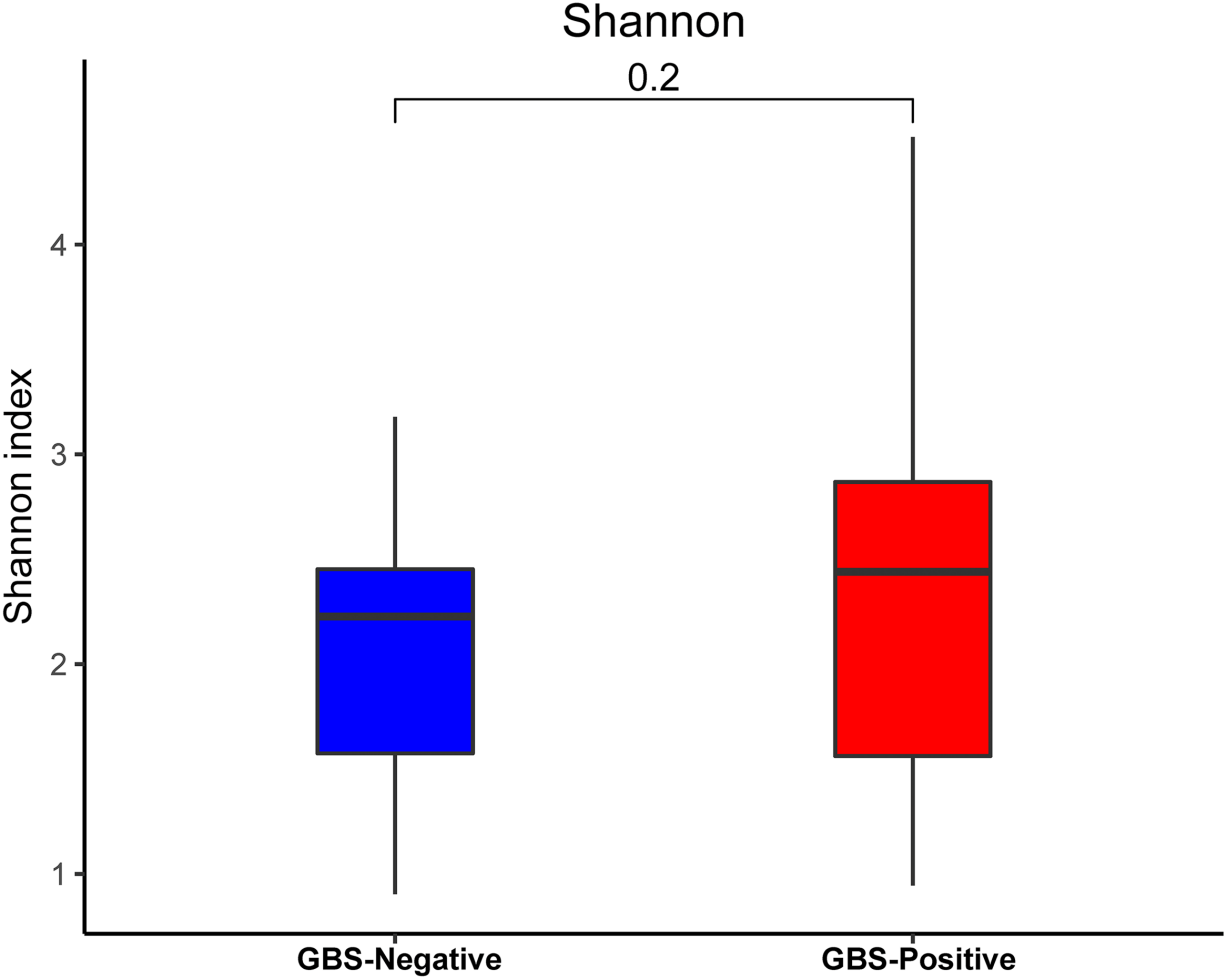
Comparison of the *α*-diversity (Shannon index) based on the OTUs profile in GBS-positive group and GBS-negative group. The *p* value was calculated by the Wilcoxon rank-sum test.

**Figure 2:**
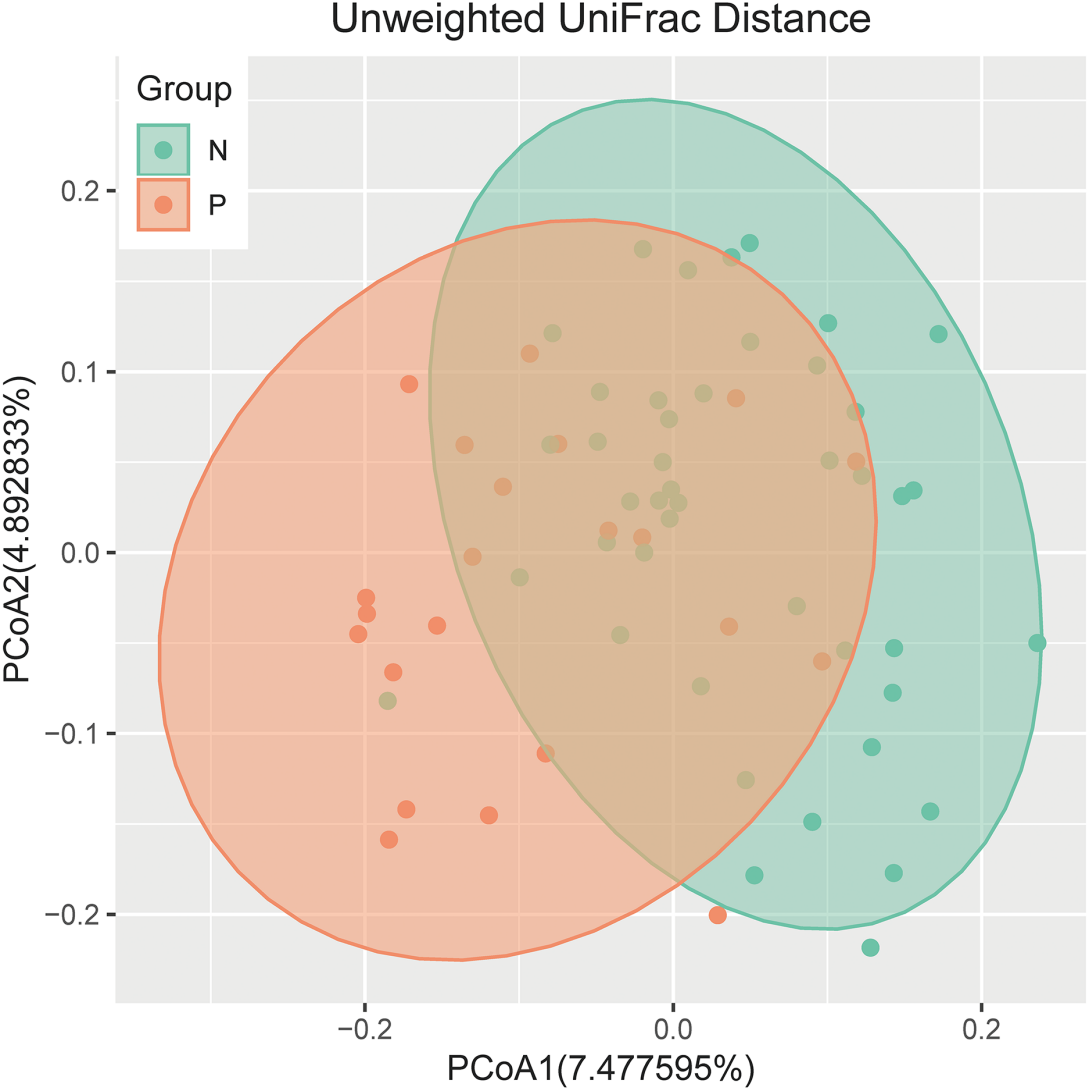
PCoA of bacterial beta diversity based on the unweighted UniFrac distance and weighted UniFrac distance. GBS-negative and GBS-positive groups are colored in green and orange, respectively.

An analysis of *alpha* diversity revealed that there was no significant differences in the Shannon index between the GBS-negative and GBS-positive groups. (Fig. 1, Wilcoxon rank sum test, *p* = 0.2). *Beta* diversity, based on the unweighted and weighted UniFrac distance, was significantly different between the two groups (*r* = 0.23, *p* = 0.001, unweighted Unifrac Distance, Fig. 2, ANOSIM).

### Relative taxon abundance in the vaginal microbiome of patients with GBS-positive and GBS-negative groups

**Figure 3:**
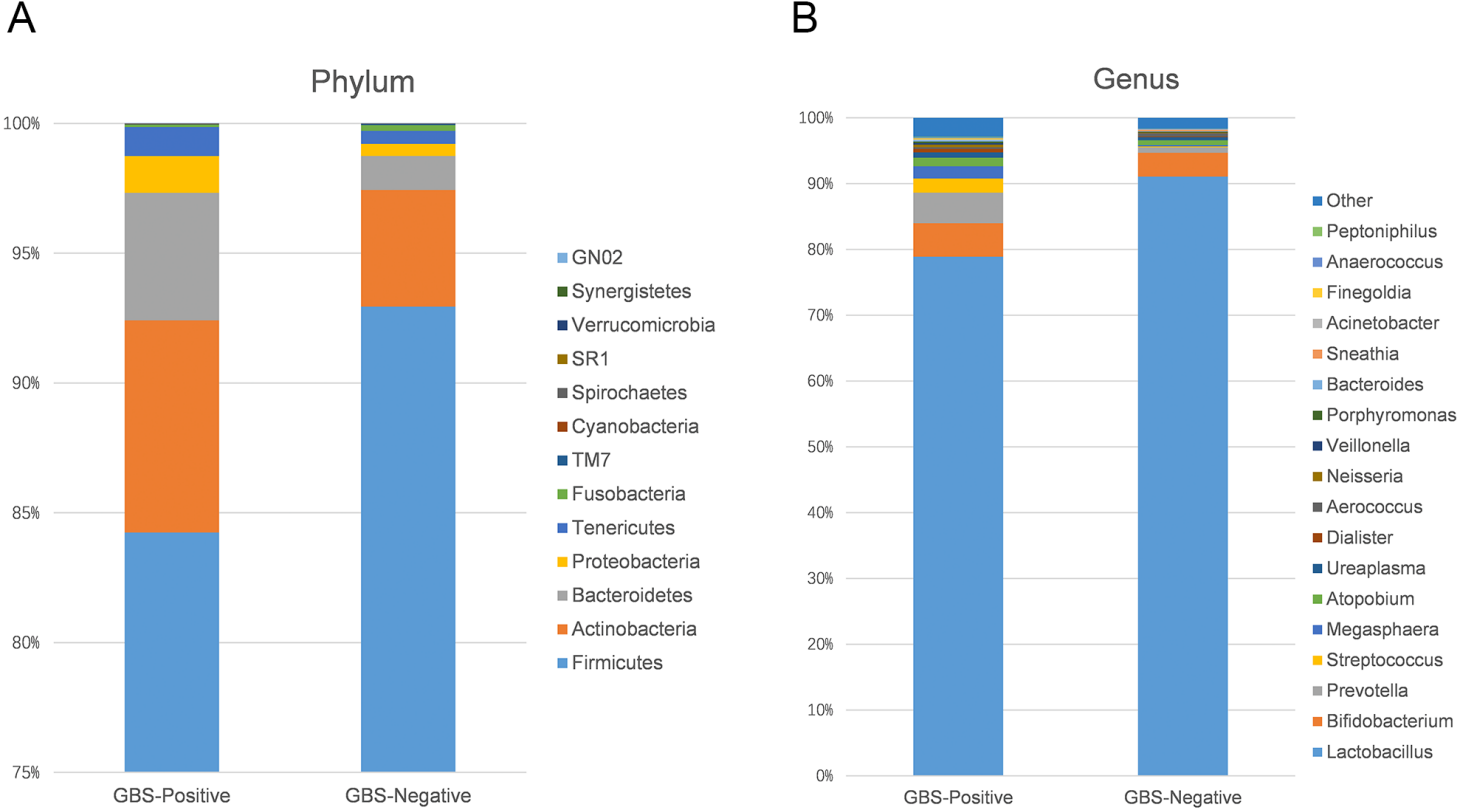
Comparison of relative taxa abundance between GBS-negative and GBS-positive groups at the phylum (A) and genus levels (B).

Using 97% as the similarity cut off, 356 qualified taxa were identified. The microbiome profiles in the vaginal samples of patients were dominated by the phylum Firmicutes (90.6% ± 16.3%), Actinobacteria (5.1% ± 13.8%), Bacteroidetes (2.6% ± 7.1%), Proteobacteria (7.5% ± 1.1%), and Tenericutes (0.6% ± 1.5%). A phylum level analysis also demonstrated that the number of Firmicutes was less abundant in patients with GBS (Fig. 3A). At the genus level, the abundances of *Prevotella*, *Megasphaera,* and *Streptococcus* were significantly higher, while *Lactobacillus* was slightly lower in the GBS-positive group compared to the GBS-negative group (Fig. 3B).

### Differential taxonomic abundance in the vaginal microbiome of patients with GBS-positive and GBS-negative groups

**Figure 4:**
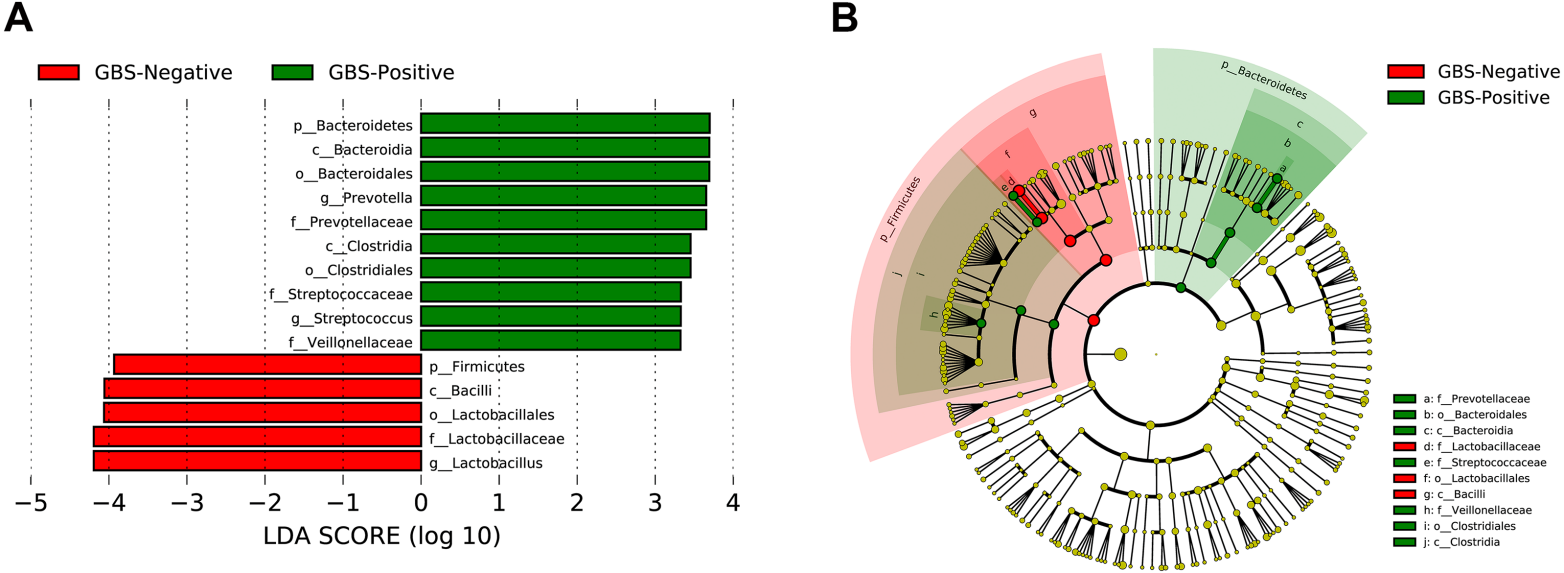
Characteristics of microbial community composition in GBS-negative and GBS-positive groups. (A) The most differentially abundant taxa between GBS-negative and GBS-positive groups (LDA score above 3) which was generated from LEfSe analysis; (B) The enriched taxa in GBS-negative and GBS-positive groups fecal microbiota were represented in Cladogram. The central point represents the root of the tree (Bacteria), and each ring represents the next lower taxonomic level (phylum to genus: p, phylum; c, class; o, order; f, family; g, genus). The diameter of each circle represents the relative abundance of the taxon.

A total of 54 taxa were found to be significantly associated with GBS, using LEfSe analysis (logarithmic discriminant analysis score >2.0). Only 15 taxa remained after filtering for those with an absolute value of the logarithmic discriminant analysis score ≥3.0 (Fig. 4). LEfSe analysis revealed a significantly higher relative abundance of some bacterial taxa in the GBS-positive group when compared with those in the GBS-negative group. These included Bacteroidetes (phylum), Bacteroidia, Clostridia (class), Bacteroidales, Clostridiales (order), Prevotellaceae, Veillonellaceae, Streptococcaceae (family), *Prevotella*, *Megasphaera,* and *Streptococcus* (genus)*,* with a significantly lower relative abundance of taxa Firmicutes (phylum), Bacilli (class), Lactobacillales (order), Lactobacillaceae (family), *Lactobacillus* (genus) in GBS-positive *vs.* GBS-negative women (*p* <0.01, Wilcoxon rank-sum test; LDA >3.0).

### Correlation of candidate bacterial taxa

**Figure 5:**
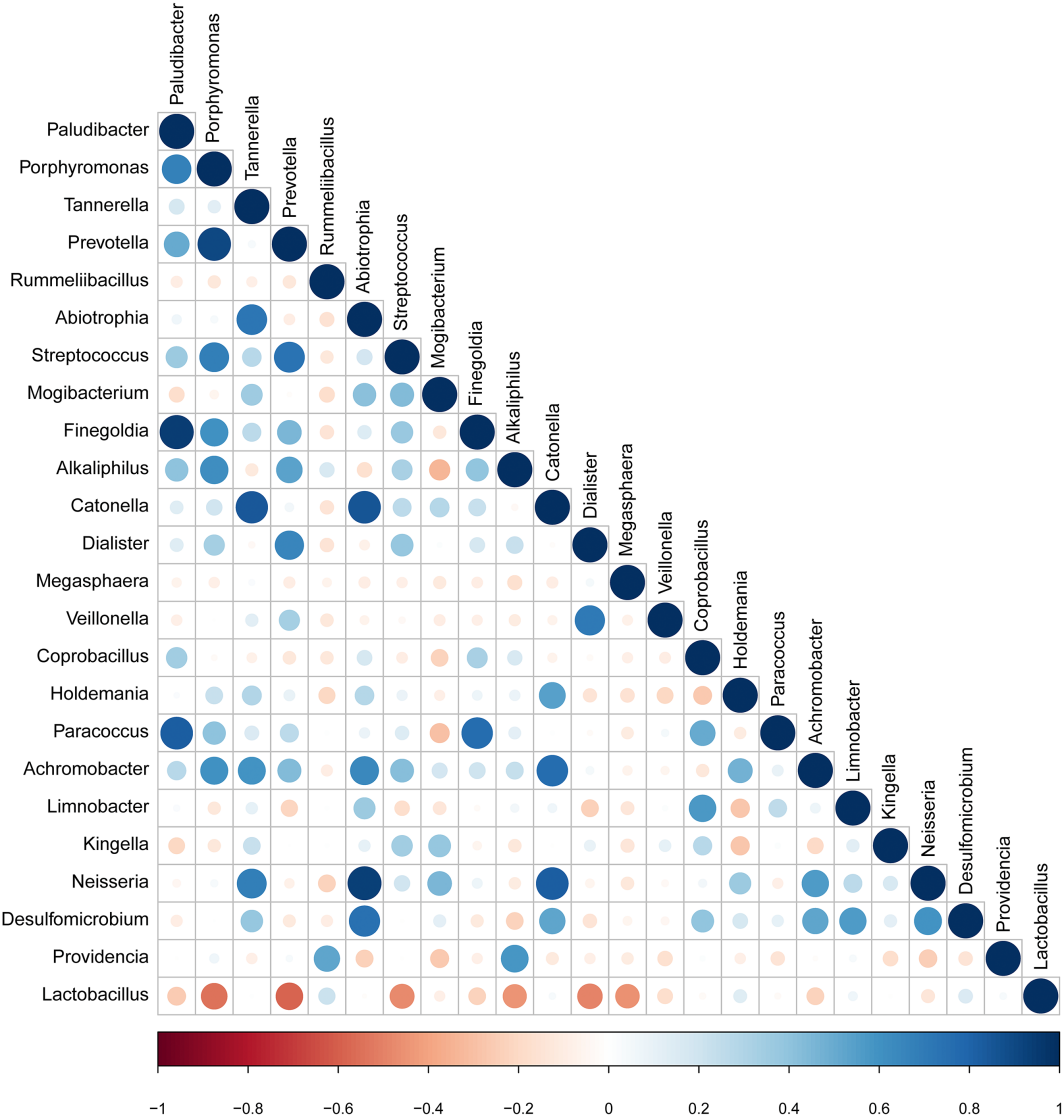
Correlation between candidate taxa. (The genus of significant difference between GBS positive and GBS negative was selected ( *p* < 0.05, LDA > 2)). There were statistically significant positive correlations between the genus Porphyromonas and Prevotella and the genus Streptococcus. While Lactobacillus showed significant negative correlations with the genus Streptococcus.

The taxa that met the threshold of significance after modeling were considered candidate bacterial taxa. Correlation between each of these candidate bacterial taxa was examined (Fig. 5). There were statistically significant positive correlations between each of the candidate taxa associated with GBS-negative status. There were statistically significant positive correlations between the genus *Porphyromonas* and *Prevotella* and the genus *Streptococcus* (*p* < 0.01). *Lactobacillus* showed significant negative correlations with the genus *Streptococcus*.

## Discussion

It is well-recognized that maternal vaginal colonization with GBS is a major risk factor for the early onset of invasive GBS disease in newborns (Centers for Disease Control and Prevention, 1996). However, the relationship between GBS and the vaginal microbiome is relatively less well-known in the Chinese cohort. The current study aimed to determine the influence of GBS colonization on Chinese women. Additionally, as an exploratory objective, and given the increasing evidence suggesting that the microbiome is an important determinant of vaginal pathogen colonization (Balkus et al., 2017; Datcu et al., 2013; Cruciani et al., 2015), the potential relationship between the composition of the vaginal microbiome and GBS colonization was analyzed in this population. It was confirmed that there is a unique microbiome in the GBS-positive group compared with the GBS-negative group. Also, the microbiome profiles of GBS-negative and GBS-positive groups were compared and microbial biomarkers were identified.

Many studies have shown that the normal vaginal microbiome is largely characterized by the dominance of a single OTU, most closely related to the *Lactobacillus* species (Ahmed et al., 2012; Albert et al., 2015; Paramel Jayaprakash, Schellenberg & Hill, 2012). The *Lactobacillus* species maintain an acidic pH in the vagina and inhibit pathogenic microorganisms (Bhandari & Prabha, 2015). The majority of healthy vaginal microbiota are dominated by *Lactobacillus*. In the current study, GBS colonization was negatively correlated with the abundance of *Lactobacillus*. An earlier study reported that there was no relationship between GBS colonization and the vaginal microbiome *alpha* diversity (Rosen et al., 2017). We were consistently unable to find a significant difference in the *alpha* diversity within the microbiome of GBS-positive and negative groups.

Individual bacterial taxa which have associations with bacterial vaginosis have had mixed associations with GBS colonization (Discacciati et al., 2011). In this study, OTUs from the genera *Prevotella, Megasphaera,* and *Streptococcus,* and OTUs from the families Prevotellaceae, Veillonellaceae, and Streptococcaceae were found to be more abundant in the GBS-positive group compared with the GBS-negative group. *Prevotella*, commonly identified in the vagina, is associated with bacterial vaginosis (BV), and has been associated with GBS-positive status (Datcu, 2014; Dols et al., 2011; Ling et al., 2010). *Megasphaera* is also associated with a GBS-positive status. Its presence can be highly correlated with BV (Datcu, 2014; Dols et al., 2011; Balkus et al., 2017; Ling et al., 2010). These complex relationships, combined with *alpha* diversity analysis and other studies suggest that GBS colonization is related to BV and to the presence or absence of specific bacterial members of the vaginal microbiome. Additionally, a decrease in *Lactobacillus* in the GBS positive group was found (Figs. 3B and 4). It has been reported that *Lactobacillus* of vaginal origin can inhibit the attachment of genital uropathogenic GBS to the vaginal epithelium (Zarate & Nader-Macias, 2006). *Lactobacillus* has also shown a significant negative correlation with the abundance of the genus *Streptococcus* (Figurec5). Furthermore, the current study indicated a statistically significant and positive correlation between the genera *Porphyromonas* and *Prevotella,* and the genus *Streptococcus*. This finding suggests that further studies need to be performed to better understand the relationship between GBS colonization and the overall vaginal microbiome community structure.

Currently, studies on the microbiome support the application of intestinal probiotic supplements for healthy intestinal immune function and microbiome balance (Dimidi, Rossi & Whelan, 2017; Nishiyama et al., 2017; Seo et al., 2017). The vaginal microbiome remains less explored than the gut microbiome although it comprises a large proportion of the female microbial network (Albert et al., 2015; Power, Quaglieri & Schulkin, 2017). Preliminary studies and data from a large health maintenance organization showed that 40–50% of GBS-colonized multiparous women were unable to receive antibiotics at least 4 h before delivery due to the rapidity of their labor (Davies et al., 2001). Most research to date has focused on the potential of probiotics to prevent bacterial vaginosis and preterm labor. The effectiveness of probiotics as a surrogate or adjunctive therapy for intrapartum antibiotic prophylaxis in GBS colonized women has rarely been evaluated (Ho et al., 2016). The findings of the current study support the idea that increasing the abundance of beneficial bacteria, such as *Lactobacillus*, can reduce GBS colonization. However, there are several limitations to the current study; for instance, the sample size was small and all patients came from a single hospital and resided in the city of Qingdao, China. Therefore, the findings may not be able to be generalized across different regions and races.

Probiotic therapies are the key to maintaining vaginal health (Bhandari & Prabha, 2015; De Gregorio et al., 2015; Power, Quaglieri & Schulkin, 2017; Surendran Nair, Amalaradjou & Venkitanarayanan, 2017; Woodman, 2016). More importantly, this potential application of probiotics needs to be investigated further in future studies in order to reduce early-onset GBS colonization and the need for antibiotic treatment during labor.

## Conclusions

In conclusion, the current study has provided preliminary data for an association between GBS colonization and the vaginal microbiome. The colonization of potential vaginal pathogens, such as *Prevotella*, *Megasphaera,* and *Streptococcus*, was significantly higher in the GBS-positive group. Thus, specific microbial taxa were associated with colonization of this significant human pathogen, highlighting a potential role for the microbiota in the promotion or inhibition of GBS colonization.

##  Supplemental Information

10.7717/peerj.7474/supp-1 Supplemental Information 1 The flow chart of this study

## Additional Information and Declarations

### Data Availability

The following information was supplied regarding data availability: All sequencing data associated with the current article are available at the NCBI SRA database (accession number: SRR8698828). Data is also available at Figshare: Zhang, Lei (2019): Vagina.fastq.gz. figshare. Dataset. https://doi.org/10.6084/m9.figshare.7857011.v1

## References

[ref-1] Ahmed A, Earl J, Retchless A, Hillier SL, Rabe LK, Cherpes TL, Powell E, Janto B, Eutsey R, Hiller NL, Boissy R, Dahlgren ME, Hall BG, Costerton JW, Post JC, Hu FZ, Ehrlich GD (2012). Comparative genomic analyses of 17 clinical isolates of Gardnerella vaginalis provide evidence of multiple genetically isolated clades consistent with subspeciation into genovars. Journal of Bacteriology.

[ref-2] Albert AY, Chaban B, Wagner EC, Schellenberg JJ, Links MG, Van Schalkwyk J, Reid G, Hemmingsen SM, Hill JE, Money D, Vogue Research Group (2015). A study of the vaginal microbiome in Healthy Canadian Women utilizing cpn60-based molecular profiling reveals distinct Gardnerella subgroup community state types. PLOS ONE.

[ref-3] Altoparlak U, Kadanali A, Kadanali S (2004). Genital flora in pregnancy and its association with group B streptococcal colonization. International Journal of Gynaecology and Obstetrics.

[ref-4] Aveyard P, Cheng KK, Manaseki S, Gardosi J (2002). The risk of preterm delivery in women from different ethnic groups. BJOG: An International Journal of Obstetrics & Gynaecology.

[ref-5] Balkus JE, Srinivasan S, Anzala O, Kimani J, Andac C, Schwebke J, Fredricks DN, McClelland RS (2017). Impact of periodic presumptive treatment for bacterial vaginosis on the vaginal microbiome among women participating in the preventing vaginal infections trial. Journal of Infectious Diseases.

[ref-6] Bhandari P, Prabha V (2015). Evaluation of profertility effect of probiotic Lactobacillus plantarum 2621 in a murine model. Indian Journal of Medical Research.

[ref-7] Brzychczy-Wloch M, Pabian W, Majewska E, Zuk MG, Kielbik J, Gosiewski T, Bulanda MG (2014). Dynamics of colonization with group B streptococci in relation to normal flora in women during subsequent trimesters of pregnancy. New Microbiologica.

[ref-8] Caporaso JG, Kuczynski J, Stombaugh J, Bittinger K, Bushman FD, Costello EK, Fierer N, Pena AG, Goodrich JK, Gordon JI, Huttley GA, Kelley ST, Knights D, Koenig JE, Ley RE, Lozupone CA, McDonald D, Muegge BD, Pirrung M, Reeder J, Sevinsky JR, Turnbaugh PJ, Walters WA, Widmann J, Yatsunenko T, Zaneveld J, Knight R (2010). QIIME allows analysis of high-throughput community sequencing data. Nature Methods.

[ref-9] Centers for Disease Control and Prevention (1996). Prevention of perinatal group B streptococcal disease: a public health perspective. Centers for Disease Control and Prevention. Recommendations and reports: morbidity and mortality weekly report. Recommendations and Reports Centers for Disease Control.

[ref-10] Cole JR, Wang Q, Cardenas E, Fish J, Chai B, Farris RJ, Kulam-Syed-Mohideen AS, McGarrell DM, Marsh T, Garrity GM, Tiedje JM (2009). The ribosomal database project: improved alignments and new tools for rRNA analysis. Nucleic Acids Research.

[ref-11] Cruciani F, Biagi E, Severgnini M, Consolandi C, Calanni F, Donders G, Brigidi P, Vitali B (2015). Development of a microarray-based tool to characterize vaginal bacterial fluctuations and application to a novel antibiotic treatment for bacterial vaginosis. Antimicrobial Agents and Chemotherapy.

[ref-12] Datcu R (2014). Characterization of the vaginal microflora in health and disease. Danish Medical Journal.

[ref-13] Datcu R, Gesink D, Mulvad G, Montgomery-Andersen R, Rink E, Koch A, Ahrens P, Jensen JS (2013). Vaginal microbiome in women from Greenland assessed by microscopy and quantitative PCR. BMC Infectious Diseases.

[ref-14] Davies HD, Miller MA, Faro S, Gregson D, Kehl SC, Jordan JA (2004). Multicenter study of a rapid molecular-based assay for the diagnosis of group B Streptococcus colonization in pregnant women. Clinical Infectious Diseases.

[ref-15] Davis RL, Hasselquist MB, Cardenas V, Zerr DM, Kramer J, Zavitkovsky A, Schuchat A (2001). Introduction of the new Centers for Disease Control and Prevention group B streptococcal prevention guideline at a large West Coast health maintenance organization. American Journal of Obstetrics and Gynecology.

[ref-16] De Gregorio PR, Juarez Tomas MS, Leccese Terraf MC, Nader-Macias ME (2015). Preventive effect of Lactobacillus reuteri CRL1324 on Group B Streptococcus vaginal colonization in an experimental mouse model. Journal of Applied Microbiology.

[ref-17] Dimidi E, Rossi M, Whelan K (2017). Irritable bowel syndrome and diet: where are we in 2018?. Current Opinion in Clinical Nutrition and Metabolic Care.

[ref-18] Discacciati MG, Simoes JA, Silva MG, Marconi C, Brolazo E, Costa ML, Cecatti JG (2011). Microbiological characteristics and inflammatory cytokines associated with preterm labor. Archives of Gynecology and Obstetrics.

[ref-19] Dols JA, Smit PW, Kort R, Reid G, Schuren FH, Tempelman H, Bontekoe TR, Korporaal H, Boon ME (2011). Microarray-based identification of clinically relevant vaginal bacteria in relation to bacterial vaginosis. American Journal of Obstetrics and Gynecology.

[ref-20] Edgar RC (2010). Search and clustering orders of magnitude faster than BLAST. Bioinformatics.

[ref-21] Ho M, Chang YY, Chang WC, Lin HC, Wang MH, Lin WC, Chiu TH (2016). Oral Lactobacillus rhamnosus GR-1 and Lactobacillus reuteri RC-14 to reduce Group B Streptococcus colonization in pregnant women: a randomized controlled trial. Taiwanese Journal of Obstetrics & Gynecology.

[ref-22] Korir ML, Manning SD, Davies HD (2017). Intrinsic maturational neonatal immune deficiencies and susceptibility to group B Streptococcus infection. Clinical Microbiology Reviews.

[ref-23] Krohn MA, Hillier SL, Baker CJ (1999). Maternal peripartum complications associated with vaginal group B streptococci colonization. Journal of Infectious Diseases.

[ref-24] Kubota T, Nojima M, Itoh S (2002). Vaginal bacterial flora of pregnant women colonized with group B streptococcus. Journal of Infection and Chemotherapy.

[ref-25] Ling Z, Kong J, Liu F, Zhu H, Chen X, Wang Y, Li L, Nelson KE, Xia Y, Xiang C (2010). Molecular analysis of the diversity of vaginal microbiota associated with bacterial vaginosis. BMC Genomics.

[ref-26] Lundelin K, Poussa T, Salminen S, Isolauri E (2017). Long-term safety and efficacy of perinatal probiotic intervention: evidence from a follow-up study of four randomized, double-blind, placebo-controlled trials. Pediatric Allergy and Immunology.

[ref-27] Magoc T, Salzberg SL (2011). FLASH: fast length adjustment of short reads to improve genome assemblies. Bioinformatics.

[ref-28] Meyer M, Kircher M (2010). Illumina sequencing library preparation for highly multiplexed target capture and sequencing. Cold Spring Harbor Protocols.

[ref-29] Nishiyama H, Nagai T, Kudo M, Okazaki Y, Azuma Y, Watanabe T, Goto S, Ogata H, Sakurai T (2017). Supplementation of pancreatic digestive enzymes alters the composition of intestinal microbiota in mice. Biochemical and Biophysical Research Communications.

[ref-30] Paramel Jayaprakash T, Schellenberg JJ, Hill JE (2012). Resolution and characterization of distinct cpn60-based subgroups of Gardnerella vaginalis in the vaginal microbiota. PLOS ONE.

[ref-31] Power ML, Quaglieri C, Schulkin J (2017). Reproductive microbiomes: a new thread in the microbial network. Reproductive Sciences.

[ref-32] Puopolo KM, Madoff LC, Eichenwald EC (2005). Early-onset group B streptococcal disease in the era of maternal screening. Pediatrics.

[ref-33] Rick AM, Aguilar A, Cortes R, Gordillo R, Melgar M, Samayoa-Reyes G, Frank DN, Asturias EJ (2017). Group B Streptococci colonization in pregnant guatemalan women: prevalence, risk factors, and vaginal microbiome. Open Forum Infectious Diseases.

[ref-34] Rosen GH, Randis TM, Desai PV, Sapra KJ, Ma B, Gajer P, Humphrys MS, Ravel J, Gelber SE, Ratner AJ (2017). Group B streptococcus and the vaginal microbiota. Journal of Infectious Diseases.

[ref-35] Segata N, Izard J, Waldron L, Gevers D, Miropolsky L, Garrett WS, Huttenhower C (2011). Metagenomic biomarker discovery and explanation. Genome Biology.

[ref-36] Seo M, Heo J, Yoon J, Kim SY, Kang YM, Yu J, Cho S, Kim H (2017). Methanobrevibacter attenuation via probiotic intervention reduces flatulence in adult human: a non-randomised paired-design clinical trial of efficacy. PLOS ONE.

[ref-37] Sundquist A, Bigdeli S, Jalili R, Druzin ML, Waller S, Pullen KM, El-Sayed YY, Taslimi MM, Batzoglou S, Ronaghi M (2007). Bacterial flora-typing with targeted, chip-based pyrosequencing. BMC Microbiology.

[ref-38] Surendran Nair M, Amalaradjou MA, Venkitanarayanan K (2017). Antivirulence properties of probiotics in combating microbial pathogenesis. Advances in Applied Microbiology.

[ref-39] Woodman Z (2016). Can one size fit all? Approach to bacterial vaginosis in sub-Saharan Africa. Annals of Clinical Microbiology and Antimicrobials.

[ref-40] Yancey MK, Duff P, Clark P, Kurtzer T, Frentzen BH, Kubilis P (1994). Peripartum infection associated with vaginal group B streptococcal colonization. Obstetrics and Gynecology.

[ref-41] Zaleznik DF, Rench MA, Hillier S, Krohn MA, Platt R, Lee ML, Flores AE, Ferrieri P, Baker CJ (2000). Invasive disease due to group B Streptococcus in pregnant women and neonates from diverse population groups. Clinical Infectious Diseases.

[ref-42] Zarate G, Nader-Macias ME (2006). Influence of probiotic vaginal lactobacilli on in vitro adhesion of urogenital pathogens to vaginal epithelial cells. Letters in Applied Microbiology.

