# Supplementary figures and images for: Group B Streptococcus colonization induces Prevotella and Megasphaera abundance-featured vaginal microbiome compositional change in non-pregnant women
Source: PeerJ. 2019 Aug 16;7:e7474. doi: 10.7717/peerj.7474 (PMC6699484; doi:10.7717/peerj.7474)

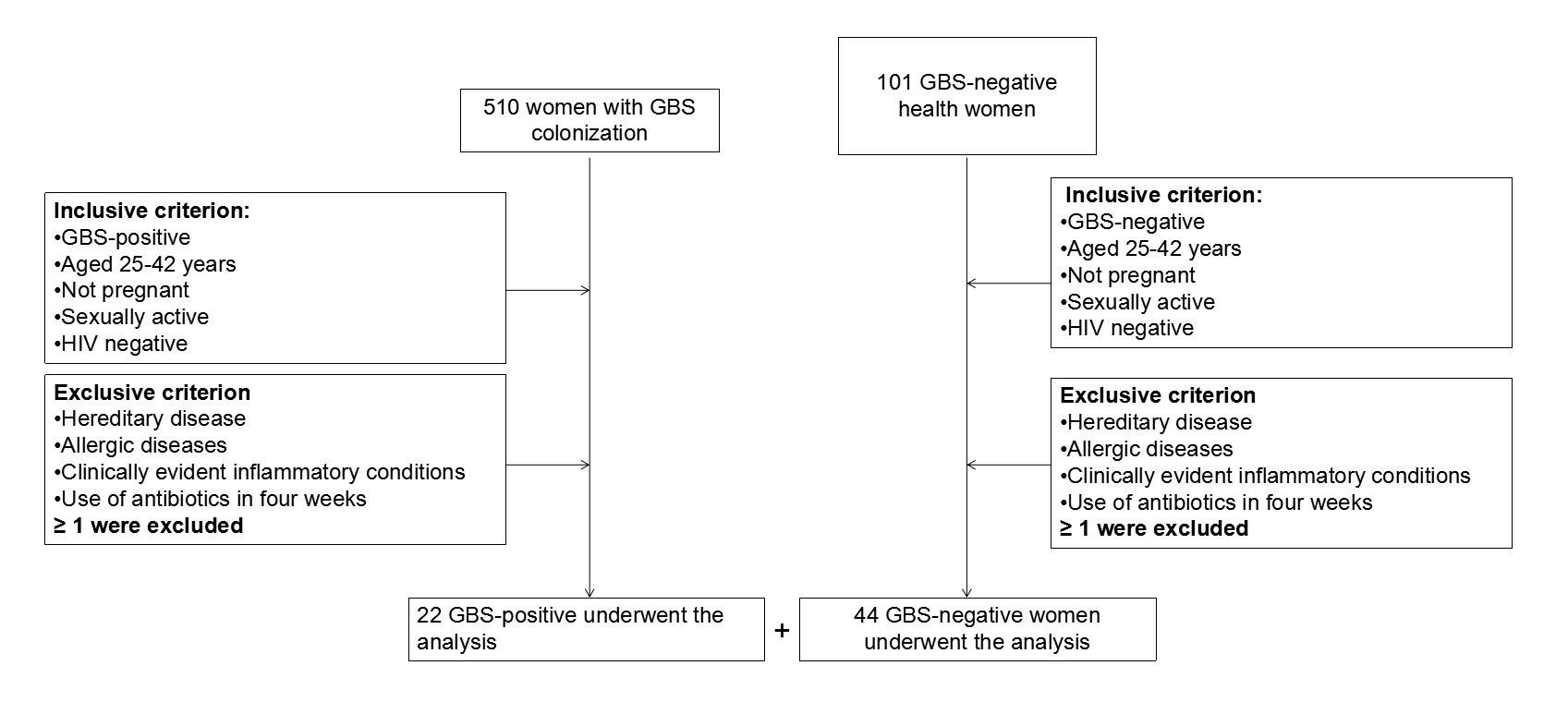

Supplement: Supplemental Information 1 [file peerj-07-7474-s001.png]
